# Extracellular vesicles produced by *Malassezia* commensal yeasts

**DOI:** 10.20517/evcna.2025.178

**Published:** 2026-05-25

**Authors:** Katarzyna Kowalik, Justyna Karkowska-Kuleta

**Affiliations:** ^1^Department of Comparative Biochemistry and Bioanalytics, Faculty of Biochemistry, Biophysics and Biotechnology, Jagiellonian University, Kraków 30-387, Poland.; ^2^Doctoral School of Exact and Natural Sciences, Faculty of Biochemistry, Biophysics and Biotechnology, Jagiellonian University, Kraków 30-387, Poland.

**Keywords:** Extracellular vesicles, *Malassezia*, MalaEx, keratinocytes, *Staphylococcus aureus*

## Abstract

*Malassezia* species were long regarded as microorganisms implicated exclusively in dermatological pathology*.* However, recent studies have further postulated a potential association between these fungi and systemic and non-cutaneous diseases, including inflammatory bowel disease, Crohn’s disease, pancreatic cancer or neurodegenerative diseases, though evidence is still preliminary. Concurrently, there is growing scientific interest in extracellular vesicles as conserved cross-kingdom mediators of intercellular communication, with the capacity to modulate host cellular responses. While extracellular vesicles have been well characterized for various microorganisms, those secreted by *Malassezia*, specified as MalaEx, remain largely underexplored to this date. This review summarizes the current knowledge of MalaEx composition and their functional significance. Detailed analyses of MalaEx have revealed that their cargo includes various enzymes, importantly aspartyl proteases, as well as allergens, with a significant enrichment of Mala s1 and Mala s7 proteins. Additionally, small RNAs were identified among the transported molecules, however, bioinformatic analyses indicated that these RNAs cannot be classified as microRNAs (miRNAs). Importantly, MalaEx have been demonstrated to interact with human immune and cutaneous cells, thereby modulating inflammatory responses. These fungal structures may also play a protective role by limiting growth of pathogenic bacteria such as *Staphylococcus aureus*, overall suggesting broader relevance of MalaEx’s functional role in regulating cutaneous microbial homeostasis and modulating the balance between pathogenic, commensal, and beneficial interactions in the skin ecosystem.

## INTRODUCTION

The genus *Malassezia* comprises lipophilic, yeast-like fungi within the phylum *Basidiomycota*^[1]^. These dimorphic microorganisms, which predominantly occur as budding yeast cells, although under certain conditions some species may also produce short hyphal elements^[2]^, were first described in 1846, with their widely recognized successful isolation and cultivation reported in 1927^[3,4]^. Then, research on *Malassezia* species continued, although early investigations were significantly constrained by methodological limitations, particularly difficulties in cultivation and in establishing appropriate growth conditions. Subsequent studies focused on resolving taxonomic classification challenges, as well as on the detailed characterization of the biological and physiological properties of *Malassezia* species and their involvement in various pathological conditions^[5]^. The nomenclatural problems, resulting in the classification of these fungi in the genus *Pityrosporum*, were largely resolved in 1995, when particular species were more precisely assigned to the genus *Malassezia* based on sequencing of the large-subunit rRNA and nuclear DNA complementarity studies^[5]^. On the basis of these analyses, seven species of *Malassezia* were defined and formally recognized, including *Malassezia furfur*, *Malassezia sympodialis*, *Malassezia obtusa*, *Malassezia globosa*, *Malassezia restricta*, *Malassezia slooffiae*, and *Malassezia pachydermatis*^[6]^; then, after 2002, next seven species of Malassezia were described, including *Malassezia dermatis*, *Malassezia equina*, *Malassezia japonica*, *Malassezia nana*, *Malassezia yamatoensis*, *Malassezia caprae*, and *Malassezia cuniculi*^[7]^. The aspects concerning the history, nomenclature, and subsequent research on *Malassezia*, involving the host immune response to fungal colonization, are comprehensively summarized in the review by Ashbee and Evans^[8]^.

First evidence suggesting an etiological role for *Malassezia* species in skin disorders such as dandruff, seborrheic dermatitis (also referred to as seborrheic eczema), and seborrheic manifestations associated with rosacea dates back to 1927^[9]^. Furthermore, over the past few decades *Malassezia* yeasts have attracted increased attention because of emerging evidence that they may also inhabit or translocate to extra-cutaneous anatomical sites or systemic compartments and influence inflammatory diseases beyond the skin^[10,11]^. Therefore, recent research has reframed *Malassezia* yeasts as opportunistic pathogens whose interactions with host immunity and bacterial microbiota could be commensal or pathogenic, depending on host genetics, immune status, and environmental factors^[12]^.

The skin mycobiome of healthy adults is typically dominated by *Malassezia* species, among which *M. restricta*, *M. globosa*, *M. sympodialis*, and *M. furfur* are the most prevalent; however, colonization dynamics, as well as species presence and abundance, vary across the lifespan^[13,14]^. The highest prevalence is observed right after birth and during puberty coinciding with increased activity of the sebaceous gland^[15]^. Most *Malassezia* species are unable to synthesize saturated fatty acids due to the absence of fatty acid synthase (FAS) genes; consequently, their unique lipid requirements have driven adaptation to sebaceous, lipid-rich microenvironments (although *M. pachydermatis* is an exception with the ability to grow on conventional culture media)^[8,16]^. Given their reliance on host-derived lipids, *Malassezia* species hydrolyze and metabolize saturated fatty acids into shorter-chain molecules, thereby influencing the composition of the cutaneous lipid environment^[12,17,18]^. Currently diagnosed dermatological syndromes associated with *Malassezia* include pityriasis versicolor, seborrheic dermatitis, dandruff, and *Malassezia*-associated folliculitis^[19,20]^. In addition, the involvement of *Malassezia* yeasts has been suggested in psoriasis and atopic dermatitis (AD), as antifungal therapy has repeatedly been reported to alleviate the severity of disease symptoms; however, the causative role of fungal presence in these conditions remains an active area of investigation^[21-24]^. Current studies have not consistently demonstrated a direct correlation between the abundance of *Malassezia* cells on the skin and the clinical severity of AD^[7,21,25-28]^; however, the increased level of anti-*Malassezia* immunoglobulin E (IgE) antibodies (sensitization) was observed to be significantly corelated with disease severity in AD patients^[29-32]^. The role of *Malassezia* in the pathogenesis of AD is hypothesized to involve an additional contribution to excessive immune cell reactivity, as alterations in skin architecture, increased transepidermal water loss, and subsequent changes in the lipid profile influence *Malassezia* colonization. Since barrier disruption enables microorganisms to penetrate deeper layers of the epidermis, thereby enhancing immune responses following exposure to fungal allergens, *Malassezia* yeasts may additionally contribute to AD pathogenesis^[21,33]^.

To date, thirteen allergens derived from *Malassezia* species have been formally recognized and included in the official allergen nomenclature. These are Mala f 2, Mala f 3 (both are peroxisomal membrane proteins) and Mala f 4 (mitochondrial malate dehydrogenase) from *M. furfur*, and from *M. sympodialis* Mala s 1, Mala s 5, Mala s 6 (cyclophilin), Mala s 7-9, Mala s 10 (heat shock protein 70), Mala s 11 (manganese superoxide dismutase), Mala s 12 [glucose-methanol-choline (GMC) oxidoreductase] and Mala s 13 (thioredoxin)^[34,35]^. However, analyses of patient sera have also revealed the presence of numerous additional antigens associated with these fungi, with varying degrees of immunological relevance^[8]^. Furthermore, species of this genus secrete a variety of enzymes, including proteases and lipases, which may be strongly involved in the modulation of host-microbial interactions^[36,37]^. An example of a protease produced by *Malassezia* is MgSAP1, secreted by *M. globosa*, which has been shown to disrupt biofilm formation by *Staphylococcus aureus*, a bacterial pathogen frequently implicated in soft tissue infections and also associated with AD^[38,39]^. MgSAP1 has been detected on the skin surface of healthy volunteers and its degradative activity against *S. aureus* protein A, which is crucial for bacterial biofilm formation, indicated that fungal-secreted protease may influence *S. aureus* pathogenicity and contribute to the balance of the human skin environment^[13,40]^. While the host may benefit from MgSAP1, its homolog MfSAP1 from *M. furfur* exhibited degradative activity against skin-associated extracellular matrix (ECM) proteins, efficiently degrading vitronectin, human epidermal keratin, thrombospondin, and fibronectin even at low enzyme concentrations^[36]^. A knockout mutant strain lacking the protease MfSAP1 exhibited reduced cell adhesion and dispersal, and subsequently caused less edema in a human 3D reconstituted epidermis model. This was later confirmed in a murine model, which demonstrated that MfSAP1 promotes inflammation^[37]^. In addition, in *M. globosa* and *M. sympodialis* several lipases, phospholipases C, and acid sphingomyelinases have been identified^[35]^.

Apart from *Malassezia* association with human skin, the role of commensal fungi in inflammatory bowel disease (IBD) has also been hypothesized. Samples extracted from sigmoid colon and cecum of patients with Crohn’s disease were significantly depleted from *Ascomycota*, with enrichment of *Basidiomycota*, especially *M. restricta* and *M. globosa*^[41]^. The polymorphism in *CARD9* (caspase recruitment domain-containing protein 9) gene encoding a signaling protein involved in antifungal immunity, recognized in IBD patients, was shown to be significantly corelated with the presence of *Malassezia*^[41]^. Studies indicate that macrophage and dendritic cell responses to *Malassezia* yeasts are mediated in part through Dectin-2 and CARD9 signaling, leading to pro-interleukin-1β (pro-IL-1β) production and activation of the NACHT, LRR and PYD domains-containing protein 3 (NLRP3) inflammasome; this highlights the importance of functional CARD9-dependent pathways in coordinating innate immune recognition of these fungi^[42,43]^. Furthermore, it has been hypothesized that conditions associated with intestinal malabsorption may create a lipid-rich environment favorable for *Malassezia* expansion, potentially linking elevated fungal colonization levels with IBD pathogenesis, although direct evidence remains limited^[44]^.

Even considering current reports and proposed hypotheses, the direct mechanisms by which *Malassezia* fungi contribute to the onset of systemic diseases have not been fully characterized, particularly with respect to yeast-host cell interactions and the mechanisms of virulence factors transfer and activity. However, growing interest in cross-kingdom communication mediated by extracellular vesicles secreted by diverse cell types has led to the hypothesis that these structures produced by *Malassezia* species may represent a mechanism underlying fungal involvement in disease development.

Extracellular vesicles produced by fungal cells were first thoroughly characterized in 2007 for the human fungal pathogen *Cryptococcus neoformans*^[45-47]^, and have been subsequently described also in other fungi, including *Candida albicans* and non-*albicans Candida* species^[48,49]^. Fungal vesicles have been identified as nano-sized structures (with a size range of 50-600 nm) capable of transporting enzymes, virulence factors, cytoplasmic proteins, toxins, pigments, quorum sensing molecules, and small RNAs across the fungal cell wall, thereby playing a critical role not only in the regulation of fungal biology, adaptation to environmental stress, biofilm formation and communication among fungi, but also in the interactions with the host^[50-52]^. Even with recent advances, numerous aspects of fungal extracellular vesicle characteristics and functionality remain to be fully elucidated^[53]^. To date, most studies have focused on the isolation and characterization of populations of extracellular vesicles, examining their size, composition, and molecular cargo. Additionally, comprehensive research has been initiated to explore their functional properties, including roles in intercellular and interkingdom communication, modulation of host immune responses, and contributions to both physiological homeostasis and pathological processes such as infection or inflammation. However, the precise regulatory pathways and factors determining cargo selection, vesicle release, and target specificity remain largely unresolved, highlighting a significant gap in our current knowledge^[47,53,54]^.

Importantly, despite increasing interest in fungal extracellular vesicles, knowledge regarding vesicles produced by *Malassezia* species remains remarkably limited. The aim of this review is therefore to consolidate and discuss the currently available evidence, highlighting the potential biological roles of these vesicles, including their interactions with host cells and with other members of the skin microbiota such as *S. aureus*.

## SIZE AND MOLECULAR COMPOSITION OF *MALASSEZIA* EXTRACELLULAR VESICLES

Extracellular vesicles secreted by *Malassezia* species were first described in 2011 in *M. sympodialis* and designated by the authors as MalaEx (*Malassezia* extracellular vesicles)^[55]^. The methodology and results concerning their characterization to date are summarized in Table 1^[55-60]^.

**Table 1:** Methods used for the analysis of extracellular vesicles derived from *Malassezia* species (MalaEx) and their basic physicochemical and molecular characterization parameters including size measurements and identification of vesicular cargo

**Species**	**Methods**	**Results**	**Reference**
Size of MalaEx			
*Malassezia sympodialis*	Growth on mDixon agar at 32 °C for 4 days, followed by 48-h culture at 37 °C in exosome-free medium TEM imaging	Diameter range: 50-200 nm Mean diameter: 100 nm	[55]
	Growth in mDixon broth supplemented with 50 mM MES (pH 6.1 and 5.5) for 48 h at 32 °C Isolation by sequential centrifugation of supernatant followed by two ultracentrifugation steps at 100,000 × *g* for 90 min Size measurement by NTA analysis and TEM imaging	Diameter range: 50-600 nm Mean diameter: 200 nm (for pH 5.5 193 nm; for pH 6.1 213.2 nm)	[56]
	Growth on mDixon agar at 32 °C for 4 days, followed by 48-h culture in RPMI 1640 medium at 37 °C and 6% CO_2_ Growth on mDixon agar at 32 °C for 4 days, followed by 72-h culture in mDixon broth at 32 °C at 200 rpm Isolation by sequential centrifugation of supernatant followed by two ultracentrifugation steps at 100,000 × *g* for 90 min Size measurement by NTA analysis with cryo-electron tomography	Diameter: 171 ± 12 nm Diameter: 245 ± 10.9 nm	[57]
	Growth for 2-4 days on mDixon agar at 32 °C, followed by 48-h culture in RPMI 1640 at 37 °C and 6% CO_2_ Isolation by sequential centrifugation of supernatant followed by overnight ultracentrifugation at 100,000 × *g* Size measurement by NTA analysis	Diameter range: 70-580 nm Mean diameter: 154 nm	[58]
*Malassezia furfur*	Growth in mDixon broth at 30 °C for 2 days Isolation by ultracentrifugation at 100,000 × *g* for 60 min Size measurement by NTA analysis and TEM imaging	Diameter range: 40-400 nm Mean diameter: 112 nm	[59]
*Malassezia restricta*	Growth in mDixon medium at 30 °C, 200 rpm/min for 48 h Isolation by ultracentrifugation at 135,000 × *g* for 3 h Size analysis with FE-SEM, FE-TEM and laser particle size analyzer	Diameter range: 50-255 nm (analyzed with FE-SEM: 50-150 nm; with FE-TEM: 100-200 nm; with laser particle size analyzer: 78-255 nm)	[60]
Composition			
*Malassezia sympodialis*	Proteins		
	Quantitative proteomics (label-free LC-MS/MS) GO enrichment and pathway analysis	Total 3,186 proteins identified of which 2,439 quantified in all replicates and 110 proteins enriched in MalaEx fraction compared to the whole cells fractions Enriched with proteins with hydrolases (i.e. lysophospholipase) and proteins with catalytic activity	[57]
	Allergens		
	Immuno-negative staining followed by immunoblotting of sucrose gradient fractions of density from 1.12 to 1.22 g/mL	Antigens on the vesicular surface	[55]
	iTRAQ analysis by LC-MS/MS	Enriched allergens: Mala s 1, Mala s 7 Additional identified allergens: Mala s 5, 6, 8-13	[57]
	Nucleic acids		
	Column-based RNA isolation followed by small RNA library preparation, size selection, and high-throughput sequencing	Small RNAs of 16-22 nucleotides	[56]

FE-SEM: Field emission scanning electron microscopy; FE-TEM: field emission transmission electron microscopy; GO: gene ontology; LC-MS/MS: liquid chromatography-tandem mass spectrometry; MES: 2-(N-morpholino) ethanesulfonic acid; mDixon: modified Dixon medium; MalaEx: Malassezia-derived extracellular vesicles; NTA: nanoparticle tracking analysis; RNA: ribonucleic acid; rpm: revolutions per minute; SEM: scanning electron microscopy; TEM: transmission electron microscopy; iTRAQ: isobaric labeling-based quantitative proteomic.

Research on extracellular vesicles produced by *Malassezia* species is relatively recent, and the available information regarding their composition and biological functions remains limited. Visualization using transmission electron microscopy (TEM)^[55,56,59]^, field-emission transmission electron microscopy (FE-TEM)^[60]^ and cryo-electron tomography^[57]^ has revealed a heterogeneous population of oval-shaped, lipid-bilayer-enclosed structures in the preparations of MalaEx, which is in line with findings reported for other fungal species^[45-54]^. TEM analysis of vesicles secreted by *M. furfur* further demonstrated variations in electron density, implying compositional differences among MalaEx^[59]^. Further size analysis of MalaEx secreted by *M. restricta* using field-emission scanning electron microscopy (FE-SEM) indicated vesicle diameters ranging from 50-150 nm, whereas FE-TEM revealed sizes of 100-200 nm^[60]^_._ Nanoparticle tracking analysis (NTA) of *M. furfur* MalaEx showed a size range of 40-400 nm, with a mean diameter of 112 nm^[59]^, which is consistent with NTA measurements of *M. sympodialis* MalaEx, revealing vesicles with diameters ranging from 70-580 nm with a mean diameter of 154 nm^[58]^. These sizes are also within the range reported for extracellular vesicles produced by other fungal species^[45-54]^.

NTA analysis performed by Johansson *et al*. further demonstrated that culture conditions influence vesicle size^[57]^. Fungal cells cultured in RPMI 1640 medium produced vesicles with a mean diameter of 171 nm, whereas MalaEx produced by *M. sympodialis* cultured for 72 h in mDixon broth had an average size of 245 nm^[57]^. After sucrose gradient purification, Gehrmann *et al*. reported vesicle sizes ranging from 50 to 200 nm for *M. sympodialis* MalaEx^[55]^. Using immunoelectron microscopy (iEM), the authors observed antigen localization on the surface of MalaEx, which was subsequently confirmed by Western blot analysis using polyclonal rabbit immunoglobulin G (IgG) against *M. sympodialis*^[55]^*.* Among the identified antigens, an approximately 70 kDa allergen was detected and shown to be recognized by IgE-containing serum from a patient with atopic eczema sensitized to *M. sympodialis*^[55]^.

Further proteomic analyses revealed that *M. sympodialis* MalaEx isolated from cells grown in mDixon broth at 32 °C for 3 days contain multiple allergens and metabolic enzymes^[55,57]^. Among the identified allergens, MalaEx were enriched in Mala s 1 and Mala s 7, both of which may contribute to the pathogenesis of inflammatory skin disorders such as AD. Mala s 1, a phosphoinositide-binding protein primarily localized to the fungal cell surface, has been suggested to participate in post-secretory modification of secondary metabolites. Mala s 7 belongs to an amplified allergen gene family. It is believed that gene duplication promotes the diversification of functions, therefore influence the virulence in *M. sympodialis*^[55,57]^. Additional allergens including Mala s 5, s 6, and s 8-13, were also identified in MalaEx but not enriched relative to cellular protein contrary to Mala s 1 and Mala s 7^[55,57]^. These findings could imply selective sorting of particular allergens to vesicles^[57]^.

Mass spectrometry-based proteomic analysis further revealed that *M. sympodialis* vesicles are enriched in enzymes associated with diverse metabolic and catalytic processes^[57]^. These include hydrolases (lysophospholipases), particularly involved in hydrolysis of sebum lipids leading to the release of fatty acids. These are thought to exert pro-inflammatory effect, contributing to skin irritation and inflammation, characteristic of *Malassezia*-associated dermatoses^[12,61]^. Importantly, *Malassezia* vesicles have been shown to contain aspartic proteinases^[12,13]^, along with carboxypeptidases, phosphatases, and glucosidases, enzymes involved in cell wall remodeling, as well as proteins associated with nucleic acid repair and metabolism^[57]^. The broad spectrum of processes in which these vesicle-associated proteins participate supports the concept that MalaEx function as molecular packages for intercellular communication^[57]^_._

Beyond proteins, the nucleic acid content of *Malassezia* extracellular vesicles has also been investigated for *M. sympodialis* grown in mDixon broth supplemented with 50 mM MES [*2*-(N-morpholino)ethanesulfonic acid] for 48 h at 32 °C^[56]^. Rayner *et al*. identified populations of small RNAs (16-22 nucleotides) within *M. sympodialis* MalaEx^[56]^. Bioinformatic analysis of the mean free energy indicated that the enriched small RNAs do not belong to the miRNA population, as they do not readily form hairpin structures, and their biogenesis is therefore suggested to occur via an independent pathway^[56]^. Previously, extracellular vesicles derived from other fungal species, such as *Cryptococcus neoformans*, *Paracoccidioides brasiliensis*, *Candida albicans*, and *Saccharomyces cerevisiae* were shown to contain predominantly RNAs shorter than 250 nucleotides, including 114 non-coding RNAs, among them snoRNAs, snRNAs, rRNAs, tRNAs, and miRNA-like sequences common to all species, as well as selected full-length mRNAs related to vesicle-mediated transport and metabolic pathways, supporting the idea that RNA-based communication may represent a shared feature among human fungal pathogens^[62]^. Importantly, further studies are required to comprehensively characterize the composition of MalaEx, particularly with regard to their DNA and lipid content, as such data remain limited.

## FUNCTIONAL STUDIES ON MALASSEZIA EXTRACELLULAR VESICLES

**Figure 1:**
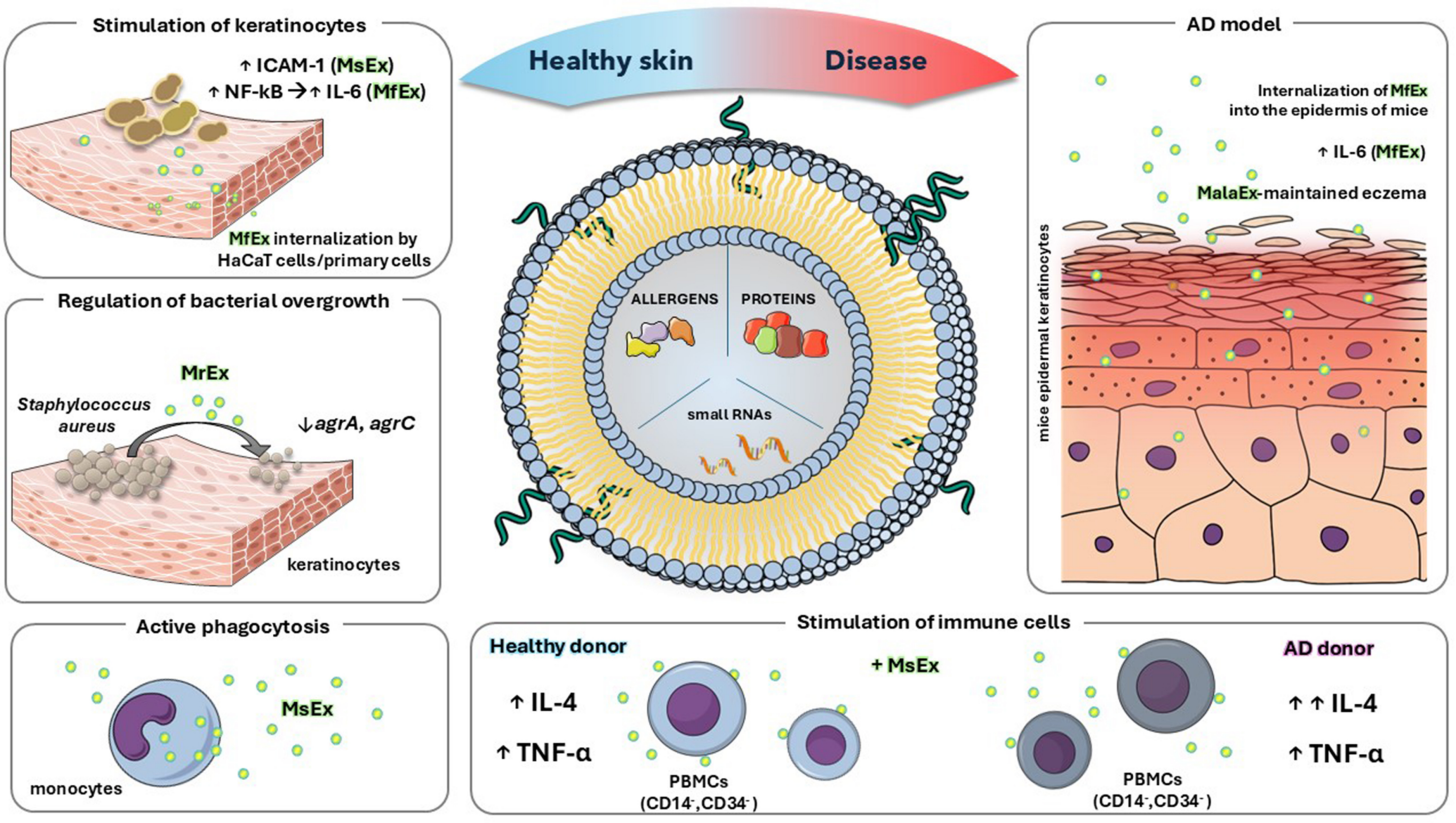
Functional characterization of *Malassezia* extracellular vesicles composition and their postulated role in healthy skin and in a model of atopic dermatitis. The figure summarizes the key conclusions of the studies described in detail in the text for extracellular vesicles released by distinct *Malassezia* species (MalaEx), including MsEx, MfEx, and MrEx. The illustration was created in the Mind the Graph Platform, available at www.mindthegraph.com. AD: Atopic dermatitis; *agr*: accessory gene regulator; IL: interleukin; TNF-α: tumor necrosis factor alpha; ICAM-1: intercellular adhesion molecule-1; NF-κB: nuclear factor kappa-light-chain-enhancer of activated B cells; MalaEx: *Malassezia*-derived extracellular vesicles; MfEx: extracellular vesicles derived from *M. furfur*; MrEx: extracellular vesicles derived from *M. restricta*; MsEx: extracellular vesicles derived from *M. sympodialis*; PBMCs: peripheral blood mononuclear cells.

Investigating the functional roles of *Malassezia* extracellular vesicles is essential for understanding their impact on host immunity and microbial interactions. Their interactions with the skin under physiological conditions, as well as their potential involvement in individuals affected by AD, have been investigated. To date, only a limited number of such reports are available; they are discussed in detail below and summarized in Figure 1.

### MalaEx secreted by Malassezia sympodialis

The molecular composition of MalaEx produced by *M. sympodialis*, including their enrichment in selected allergens, enzymes, proteins, and small RNAs, suggests a role in cross-kingdom communication with both host cells and the surrounding microbial niche. In microbially enriched regions of the skin, keratinocytes, the predominant epidermal cell type, together with resident immune cells, are profoundly modulated by metabolites secreted by commensal and pathogenic microorganisms^[63]^. Studies performed with fluorescence and super-resolution microscopy have demonstrated that MalaEx can adhere to human cells and are actively internalized by human keratinocytes and monocytes, as vesicle uptake occurred only at the physiological temperature of 37 °C and was not observed at 4 °C; based on these observations, the authors suggested that MalaEx are likely internalized via endocytosis^[57]^. Following uptake, vesicles exhibited a tendency for localization within the cytoplasm and near nuclei, indicating that their cargo may exert intracellular effects. Exposure of MalaEx at a concentration of 10 µg/mL to primary human keratinocytes obtained from skin donors induced the upregulation of intercellular adhesion molecule-1 (ICAM-1), a surface molecule essential for leukocyte adhesion and trafficking^[58]^. The enhancement varied between biological replicates, with approximately 5%-22% induction observed, consistent with the positive control of LPS, which showed a mean induction of 26% ± 3.6%. Interestingly, *M. sympodialis* yeast cells (0.6 × 10^5^ and 3 × 10^5^ cells/well) appeared to have a lower activating potential, as ICAM-1 upregulation ranged from 8%-15%, suggesting that MalaEx may be involved in the initiation or amplification of immune responses and thus contribute to microbe-host cross-talk^[58]^.

Similarly, *M. sympodialis* extracellular vesicles have been shown to modulate cytokine production by the immune cells. Gehrmann *et al*. demonstrated that MalaEx, administered at protein concentrations of 23 and 230 ng, enhanced interleukin-4 (IL-4) production in cluster of differentiation 14/cluster of differentiation 34 (CD14/CD34)-depleted peripheral blood mononuclear cells (PBMCs) from healthy controls in a dose-dependent manner compared to the medium control^[55]^. MalaEx also induced a dose-dependent tumor necrosis factor α (TNF-α) response at both concentrations in healthy donors. This impact was further investigated by examining whether antigens presented on host endogenous vesicles from monocyte-derived dendritic cells (MDDCs) contacted with yeast cells could exert a similar effect. Interestingly, vesicles obtained from co-culture of MDDCs with *M. sympodialis* were able to significantly induce IL-4 and TNF-α production in CD14/CD34-depleted PBMCs from healthy individuals, although the effect was observed when vesicles were applied at a higher protein concentration of 4 µg^[55]^.

Similarly, *M. sympodialis* extracellular vesicles have been shown to modulate cytokine production by the immune cells. Gehrmann *et al*. demonstrated that MalaEx, administered at protein concentrations of 23 and 230 ng, enhanced interleukin-4 (IL-4) production in cluster of differentiation 14/cluster of differentiation 34 (CD14/CD34)-depleted peripheral blood mononuclear cells (PBMCs) from healthy controls in a dose-dependent manner compared to the medium control^[55]^. MalaEx also induced a dose-dependent tumor necrosis factor α (TNF-α) response at both concentrations in healthy donors. This impact was further investigated by examining whether antigens presented on host endogenous vesicles from monocyte-derived dendritic cells (MDDCs) contacted with yeast cells could exert a similar effect. Interestingly, vesicles obtained from co-culture of MDDCs with *M. sympodialis* were able to significantly induce IL-4 and TNF-α production in CD14/CD34-depleted PBMCs from healthy individuals, although the effect was observed when vesicles were applied at a higher protein concentration of 4 µg^[55]^.

### MalaEx produced by Malassezia furfur

While *M. sympodialis* has been the most extensively studied species, similar effects have been observed for vesicles derived from *M. furfur*^[59]^. Internalization of *M. furfur* MalaEx by keratinocytes was demonstrated *in vitro* using the HaCaT cell line, as well as *in vivo* in intact murine epidermis, where vesicles were shown to penetrate the entire epidermal layer^[59]^. Due to the lack of internalization of free DiI dye, the authors speculate that the vesicles are internalized via phagocytosis. Exposure to MalaEx administered at a sterol concentration of 500 ng for 1 h stimulated the release of interleukins interleukin-6 (IL-6) and IL-1β. Additional time-course analyses demonstrated that sustained upregulation of cytokine expression was observed exclusively for IL-6, whereas IL-8 mRNA levels did not differ significantly from those in unstimulated keratinocytes^[59]^. Additionally, after topical application of MalaEx on murine skin, an interesting observation was made, as vesicles preferentially localized in close proximity to hair follicles. The activation of IL-6 was also shown to be driven through the NF-κB signaling pathway, a central regulator of inflammation processes. The induction of IL-6 by *M. furfur* vesicles underscores the capacity of MalaEx to activate innate immune signaling pathways and supports the hypothesis of the initiation of inflammatory cascades even in the absence of ongoing infection^[59]^.

### Role of MalaEx in AD

The release of vesicles by *Malassezia* species, which contain allergens, enzymes, and surface-presented antigens, may contribute to the pathogenesis of dermatological conditions associated with *Malassezia* colonization. Due to their direct interaction with the skin, most studies investigating the effects of MalaEx have focused on AD. MalaEx have been shown to exert significant immunomodulatory effects on keratinocytes in healthy skin, suggesting that the physiological or pathological context may influence the observed outcomes^[57,58]^. Studies conducted on extracellular vesicles released by other fungal species, such as *Candida albicans* and *Fonsecaea* species indicate that stress conditions such as oxidative stress or nutrient depletion could impact the characteristics of produced vesicles, their morphology, size, concentration or proteomic profile^[64-66]^. These alterations may also contribute to the pathogenesis of conditions associated with MalaEx production. To investigate the potential impact of pH on MalaEx production, Rayner *et al*. examined extracellular vesicles produced by *M. sympodialis* cultured at pH values corresponding to normal skin and the elevated pH observed in AD patients^[56]^. Interestingly, elevated pH conditions associated with the pathogenic state had no significant impact on the concentration, size, morphology, or molecular content of MalaEx secreted by *M. sympodialis*. The results showed no statistically significant differences in protein concentration between MalaEx samples, and similarly, no biological differences were observed in the expression of small RNA features^[56]^. Therefore, these results indicate that elevated pH has no significant effect on MalaEx production *in vitro*; however, the literature provides no information regarding the concentration of secreted vesicles under *in vivo* conditions.

Under inflammatory states associated with skin disorders, the epidermal barrier becomes more permeable, allowing MalaEx to penetrate and diffuse more effectively into the dermal layers. This increased access enables vesicles enriched with allergens to come into contact with cells involved in the innate immune response^[59]^. A concentration of 23 ng of MalaEx derived from *M. sympodialis* triggered increased production of IL-4 in CD14/CD34-depleted PBMCs, with upregulation being more pronounced in AD patients compared to healthy individuals. This trend was observed for both MalaEx and whole *M. sympodialis* fungal cells. Additionally, MalaEx were shown to induce TNF-α expression, although no significant differences were observed between AD patients and healthy controls^[55]^. The authors suggest that MalaEx may elicit different immune responses than whole yeast cells because immunogenic molecules are concentrated on the vesicle surface, potentially altering host-microbe interactions and allergenicity^[55]^. These studies further demonstrated that following internalization of fungal cells MDDCs produced their own vesicles, on the surface of which *Malassezia* antigens were detected (referred to by the authors as DCexo Mala)^[55]^. *Malassezia* antigens were thus found not only on the surface of MalaEx but also on vesicles secreted by MDDCs after rapid internalization of fungal cells^[55]^. Stimulation of PBMCs with these vesicles (quantified by protein content and applied at 4 µg) led to enhanced IL-4 production, with a stronger response observed in AD patients, suggesting increased sensitivity to *Malassezia* allergens under pathological conditions^[55]^.

### Host-microbe communication including Staphylococcus aureus and Malassezia restricta extracellular vesicles

Current studies have mainly focused on understanding the impact of MalaEx on host cells and their potential role in dermatitis development. *Malassezia* species inhabit dry, moist, and sebaceous skin environments, coexisting with distinct microbial communities specific to each region. Therefore, investigating the role of secreted vesicles in cross-kingdom communication is crucial for elucidating regulatory mechanisms that control pathogen overgrowth, which is particularly relevant in skin pathogenesis involving biofilm-forming bacteria such as *S. aureus* and *Staphylococcus epidermidis*^[67,68]^. The interaction between MalaEx derived from *M. restricta* and *S. aureus* has been explored experimentally, with studies showing that 10-100 μg/mL of extracellular vesicles from *M. restricta* can suppress *S. aureus* proliferation and biofilm formation, providing one of the first examples of *Malassezia* extracellular vesicle-bacterial interplay on healthy skin^[60]^. Although staphylococcal skin infections are common across dry, moist, and sebaceous skin regions, bacterial cells were detected in lower quantities compared to *M. restricta* cells. Fungal cells were found to be significantly more abundant on the cheek, alar crease, auditory canal, and retroauricular crease, whereas *S. aureus* predominated only on the toenails^[21]^. This report demonstrated that MalaEx derived from *M. restricta* exert an inhibitory effect on bacterial growth, which was particularly pronounced after 11 h of incubation^[60]^. The presence of fungal vesicles reduced bacterial cell proliferation and was associated with downregulation of the quorum-sensing genes *agrA* and *agrC*, which are part of the *S. aureus* accessory gene regulator (agr) system: AgrC acts as a membrane-bound sensor kinase detecting autoinducing peptides, while AgrA functions as the response regulator controlling expression of virulence factors^[69]^. Metabolic changes also affected cell surface structures. FE-SEM imaging of the bacteria treated with 100 μg/mL of MalaEx revealed a significantly smoother bacterial surface compared with the irregular surface of the untreated group^[60]^. The authors hypothesized that the uneven surface of untreated cells is related to the presence of anchored proteins on the cell wall^[60]^. These findings may also indicate that MalaEx modulates the surface exposure of cell wall-associated proteins, suggesting a potential mechanism by which fungal extracellular vesicles influence host-microbe interactions.

## FUTURE PERSPECTIVES

The enrichment of major allergens within MalaEx provides a possible mechanism for antigen delivery and sensitization. Because secreted vesicles are of nanometer size, they can diffuse through the stratum corneum more easily than intact yeast cells, allowing allergens to reach antigen-presenting cells in the epidermis and dermis. The activation of keratinocytes and monocytes suggests engagement of pattern recognition receptors such as Toll-like receptor 2 (TLR2), Toll-like receptor 4 (TLR4), and Dectin-1, all of which are known to detect fungal components^[43,70,71]^. Cell wall components could serve as ligands for these receptors, initiating downstream signaling and cytokine production. In other fungal species, vesicle-derived small RNAs have been shown to interact with host cells and subsequently modulate host immunity^[72,73]^. However, the functional impact of small RNAs contained in MalaEx has not yet been demonstrated. Future studies are therefore warranted to further characterize the nucleic acid cargo of MalaEx produced by different *Malassezia* species. Despite significant advances, the precise cellular targets of MalaEx and their uptake pathways remain incompletely understood, representing a compelling area for future investigation. As in other fungi, the biogenesis of extracellular vesicles remains insufficiently characterized, and the precise molecular mechanisms and environmental factors that influence this process have yet to be fully elucidated, which is particularly relevant in the context of pathogenic microorganisms.

Due to the significant enrichment of allergens and surface-presented antigens, MalaEx may serve as potential biomarkers in studies of fungal dermatitis, where quantitative profiling of specific vesicular components could improve disease diagnosis and monitoring of disease progression. Furthermore, as explored in the literature for vesicles derived from other organisms^[74-76]^, *Malassezia*-derived vesicles could be investigated as vaccine candidates or as delivery systems for bioactive molecules, particularly in the context of topical therapies. In addition, to comprehensively investigate *Malassezia* interactions with skin cells, diverse immune cell types, and the resident skin microbiota, and to accurately characterize the resulting immune responses, the development of physiologically relevant host-pathogen infection models is required, including systems that incorporate extracellular vesicles produced by all interacting members within the studied environment. In addition, it is important to emphasize that findings derived from murine models, especially concerning immune responses and skin architecture, may not fully recapitulate human physiology and should be interpreted with appropriate caution.

## CONCLUSIONS

Although data on extracellular vesicles secreted by *Malassezia* species remain limited, current studies indicate that MalaEx have a significant impact on both the resident microbial community and the host. These vesicular carriers have been shown to contain proteins, enzymes, allergens, lipids, and small RNAs, which collectively influence skin physiology and immune responses. Functionally, MalaEx modulate keratinocyte activation and stimulate cytokine release from immune cells. Moreover, they appear to affect the bacterial-fungal balance on human skin, suggesting a potential role in preventing microbial overgrowth.

## DECLARATIONS

### AI and AI-assisted tools statement

During the preparation of this manuscript, the AI tool ChatGPT (version GPT-5-mini, released 2023-11-06) was used solely for language editing. The tool did not influence the study design, data collection, analysis, interpretation, or the scientific content of the work. All authors take full responsibility for the accuracy, integrity, and final content of the manuscript.

### Copyright

© The Author(s) 2026
